# Different lactic acid bacteria and their combinations regulated the fermentation process of ensiled alfalfa: ensiling characteristics, dynamics of bacterial community and their functional shifts

**DOI:** 10.1111/1751-7915.13785

**Published:** 2021-03-05

**Authors:** Jie Bai, Zitong Ding, Wencan Ke, Dongmei Xu, Museng Wang, Wenkang Huang, Yixin Zhang, Fang Liu, Xusheng Guo

**Affiliations:** ^1^ State Key Laboratory of Grassland Agro‐ecosystems School of Life Sciences Lanzhou University Lanzhou 730000 China; ^2^ State Key Laboratory of Grassland Agro‐ecosystems College of Pastoral Agriculture Science and Technology Lanzhou University Lanzhou 730020 China; ^3^ Probiotics and Biological Feed Research Centre Lanzhou University Lanzhou 730000 China

## Abstract

The objectives of this study were to investigate the adaptation and competition of *Lactobacillus plantarum*, *Pediococcus pentosaceus* and *Enterococcus faecalis* inoculated in alfalfa silage alone or in combination on the fermentation quality, dynamics of bacterial community, and their functional shifts using single‐molecule real‐time (SMRT) sequencing technology. Before ensiling, alfalfa was inoculated with *L. plantarum* (Lp), *P. pentosaceus* (Pp), *E. faecalis* (Ef) or their combinations (LpPp, LpEf, LpPpEf) and sampled at 1, 3, 7, 14 and 60 days. After 60‐days fermentation, the Lp‐, Pp‐ and LpPp‐inoculated silages had lower pH but greater concentrations of lactic acid were observed in Pp, LpEf and LpPpEf‐inoculated silages. The inoculants altered the keystone taxa and the bacterial community dynamics in different manners, where *L. plantarum*, *Weissella cibaria* and *L. pentosaceus* dominated the bacterial communities after 14 days‐fermentation in all treatments. The silages with better fermentation quality had simplified bacterial correlation structures. Moreover, different inoculants dramatically changed the carbohydrate, amino acid, energy, nucleotide and vitamin metabolism of bacterial communities during ensiling. Results of the current study indicate that effect of different inoculants on alfalfa silage fermentation was implemented by modulating the succession of bacterial community, their interactions and metabolic pathways as well during ensiling.

## Graphical abstract

This study were investigated the adaptation and competition of *Lactobacillus plantarum*, *Pediococcus pentosaceus*, and *Enterococcus faecalis* inoculated in alfalfa silage alone or in combination on the fermentation quality, dynamics of bacterial community, and their functional shifts of alfalfa silage. The inoculants altered the keystone taxa, dynamics of bacterial community and their functions in different manners, and the silages with better fermentation quality had simplified bacterial correlation structures.

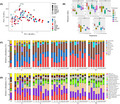

## Introduction

Ensiling is a method of preserving high‐quality forage, which is initiated by a complex microbial community under anaerobic environments. During the initial fermentation process, the lactic acid bacteria (LAB) abundance normally increases quickly and subsequent bacterial communities tend to be established, which is crucial for later fermentation and final silage quality (Yang *et al*., 2019). McAllister *et al*. (2018) reported that the microbial compositions of alfalfa silage were more diverse than that of cereal silages, which often caused the growth of undesirable microorganisms during ensiling. Inoculating LAB is one of the most common methods of improving silage quality because it can directly increase the abundance of beneficial bacteria and accelerate the fermentation of lactic acid. However, LAB inoculants perform different functions and have different fermentation patterns during ensiling. Lactic acid‐producing cocci (*Leuconostocs*, *Pediococcus*, *Lactococci* and *Enterococci*) initiate lactic fermentation at the early ensiling process, while these genera had lower tolerance to low pH than *Lactobacillus* (Ni *et al*., 2017; Yang *et al*., 2019). Lactic acid‐producing rod (*Lactobacillus*) plays an important role for pH reduction at the later stage (Ni *et al*., 2017). In addition, Langston and Bouma (1960) reported that better quality silages were obtained when the early bacterial flora consistent predominantly of cocci. Most cocci, expect the *Pediococci*, disappeared a few days after ensiling. Therefore, inoculation of *Lactobacillus plantarum*, *Pediococcus pentosaceus* and *Enterococcus faecalis* alone or their combinations may have different effects on the fermentation process of silage.

In recent years, the PacBio sequencing, in conjunction with SMRT (single‐molecule real‐time) technology has been employed to track the changes in microbial communities and identify dominant species in ensiled forages because it can be used to reveal the bacterial profile of target samples at the species level (Guo *et al*., 2018; Xu *et al*., 2019; Xu *et al*., 2020). Recent studies have focused not only on changes in microbial diversity, but also on microbial interaction and functional prediction (Wang *et al*., 2019a; Xu *et al*., 2020). The microbial interactions are crucial for successful establishment and maintenance of silage starter cultures during ensilage. In addition, many metabolites are produced by LAB during fermentation (Sun *et al*., 2012). Therefore, a better understanding of microbial metabolic pathways underlying silage fermentation could provide us important bioinformation to regulate silage fermentation.

To date, numerous studies have evaluated the effects of different inoculants on silage fermentation. However, most of these studies just focused on the changes of fermentation characteristics and chemical compositions of silage without exploring the complex microbial community succession, microbial interactions and their functional shifts. These biological processes may have different responses to different silage inoculants. Therefore, the objectives of this study were to investigate the effect of *L. plantarum*, *P. pentosaceus* and *E. faecalis* inoculated in alfalfa silage alone or in combination on the fermentation quality, dynamics of bacterial community, and their functional shifts, and to figure out modulation of different lactic acid bacteria on microbial interactions in ensiled alfalfa.

## Results and discussion

### Chemical and fermentation characteristics of alfalfa silage during ensiling

The chemical and fermentation characteristics of alfalfa silage during ensiling are listed in Tables 1 and 2. Overall, inoculants had significant impacts on pH value, amounts of lactic, acetic, and propionic acids, and ratio of lactic acid to acetic acid (LA/AA) in the same ensiling day (*P* < 0.05, Table 1). After 1 days of ensiling, different degrees of lactic acid fermentation were initiated among the groups, resulting in various rates of lactic acid production and pH reduction. The most rapid decrease in pH and increase in lactic acid concentration were observed in the Pp‐inoculated silage, followed by Lp‐, and LpPp‐inoculated silages during the early stage of ensiling. These results indicated that Pp was the most efficient initiator of fermentation during the early stage of ensiling, consistent with the report of Liu et al. (2019). A slow reduction in pH and lower lactic acid concentrations was found in the control, Ef‐, LpEf‐ and LpPpEf‐inoculated silages in the early stage of ensiling. It is known that cocci‐type LABs dominate fermentation at the early stage of ensiling and result in a quick decrease of silage (McDonald *et al*., 1991). However, the silages inoculated with Ef alone or in combination with Lp or LpPp did not show an ability to lower the pH as rapidly as the Pp‐inoculated silage. It might be due to the poor activity of Ef that was not enough to compete with the other epiphytic microorganisms. At the end of ensiling, a lower pH was also found in Pp‐, Lp‐ and LpPp‐inoculated silages. However, interestingly, higher lactic acid concentrations were found in Pp‐, LpEf‐ and LpPpEf‐inoculated silages. It might be due to a higher concentration of NH_3_‐N in silages inoculated with LpEf or LpPpEf neutralized the acid, and different organic acid metabolisms occurred in present silages. In addition, a lower lactic acid concentration and a higher pH value were observed in the Ef‐inoculated silage as compared with the control during the entire ensiling procedure (*P* < 0.05). This agrees well with the report of Cai (1999) that inoculation with *Enterococci* did not promote silage fermentation.

**Table 1:** Fermentation characteristics of alfalfa silage after 60 days of ensiling

Item	Treatment^a^	D^b^					SEM^c^	*P* value^d^		
		1	3	7	14	60		T	D	T × D
pH	C	5.16	5.15	5.23	5.23	5.03	0.015	<0.001	<0.001	<0.001
	Lp	5.02	5.06	5.09	5.08	4.86				
	Pp	4.87	4.93	4.96	5.02	4.81				
	Ef	5.21	5.27	5.20	5.28	5.13				
	LpPp	5.05	5.04	5.01	5.02	4.88				
	LpEf	5.25	5.22	5.10	5.15	5.04				
	LpPpEf	5.19	5.25	5.09	5.17	5.06				
Lactic acid(g kg DM^‐1^)	C	43.8	41.6	46.3	55.6	75.9	1.082	<0.001	<0.001	<0.001
	Lp	63.5	52.4	52.1	65.5	97.3				
	Pp	76.8	54.3	76.4	99.1	109				
	Ef	34.8	34.5	40.7	48.9	67.8				
	LpPp	49.3	56.9	55.5	84.4	97.1				
	LpEf	35.6	37.5	42.6	84.9	117				
	LpPpEf	33.5	47.7	46.3	88.6	103				
Acetic acid(g kg DM^‐1^)	C	45.2	47.9	37.2	41.6	42.3	0.958	0.023	<0.001	<0.001
	Lp	54.0	37.9	29.2	37.7	35.2				
	Pp	55.3	20.2	48.4	52.2	38.6				
	Ef	51.4	31.9	38.7	43.1	47.1				
	LpPp	54.0	14.5	30.0	36.1	32.6				
	LpEf	42.3	33.0	31.1	45.4	61.4				
	LpPpEf	45.9	43.5	43.4	46.5	55.8				
Propionic acid(g kg DM^‐1^)	C	1.46	3.90	5.28	9.35	19.6	0.169	<0.001	<0.001	<0.001
	Lp	2.99	2.53	3.33	8.25	18.0				
	Pp	3.86	1.62	4.14	9.18	21.4				
	Ef	2.25	3.30	5.36	10.46	17.6				
	LpPp	2.35	1.63	2.37	6.78	15.4				
	LpEf	2.59	3.28	3.11	9.31	23.1				
	LpPpEf	2.42	4.06	5.73	9.46	22.3				
Lactic acid/acetic acid	C	0.97	0.87	1.24	1.34	1.80	0.054	<0.001	<0.001	<0.001
	Lp	1.18	1.38	1.78	1.74	2.76				
	Pp	1.39	2.71	1.58	1.90	2.84				
	Ef	0.68	1.09	1.05	1.13	1.44				
	LpPp	0.91	3.91	2.00	2.34	2.98				
	LpEf	0.84	1.14	1.37	1.87	1.91				
	LpPpEf	0.73	1.10	1.07	1.91	1.85				

^a^ C, control, no additive; Lp, *L. plantarum*; Pp, *P. pentosaceus*; Ef, *E. faecalis*; LpPp, combination of *L. plantarum* and *P. pentosaceus*; LpEf, combination of *L. plantarum* and *E. faecalis*; LpPpEf, combination of *L. plantarum*, *P. pentosaceus*, and *E. faecalis*.

^b^ D, fermentation time (days).

^c^ SEM, standard error of the mean.

^d^ T, inoculants treatment; D, fermentation time (d); T × D, the interaction between inoculants treatment and fermentation time.

**Table 2:** Chemical composition of alfalfa silage after ensiling for 60 days

Item	Inoculations							SEM	*P*‐value
	C	Lp	Pp	Ef	LpPp	LpEf	LpPpEf		
DM, g kg FM^‐1^	310^ab^	315^a^	308^ab^	306^b^	305^b^	303^b^	296^c^	1.344	<0.001
DM loss, g kg^‐1^	47.5^ab^	43.8^b^	42.3^b^	49.6^ab^	44.3^b^	49.0^ab^	58.7^a^	1.343	0.007
WSC, g kg DM^‐1^	11.2	11.2	11.6	11.3	11.5	11.6	11.3	0.067	0.354
CP, g kg DM^‐1^	225^ab^	226^ab^	234^a^	228^ab^	234^a^	227^ab^	222^b^	1.179	0.008
NPN, g kg total N^‐1^	507^a^	440^cd^	425^de^	478^ab^	399^e^	465^bc^	469^bc^	7.764	<0.001
NH_3_‐N, g kg total N^‐1^	123^abc^	116^c^	117^bc^	130^a^	116^c^	125^ab^	127^a^	1.283	<0.001
aNDF, g kg DM^‐1^	334^a^	324^a^	332^a^	301^b^	299^b^	304^b^	297^b^	3.532	<0.001
ADF, g kg DM^‐1^	250^a^	248^ab^	247^ab^	235^bc^	229^c^	229^c^	227^c^	2.227	<0.001

ADF, acid detergent fibre; aNDF, neutral detergent fibre (aNDF assayed with a heat‐stable amylase and expressed inclusive of residual ash) ; CP, crude protein; DM loss, dry matter loss; DM, dry matter; NH_3_‐N, ammonia nitrogen; NPN, non‐protein nitrogen; SEM, standard error of the mean; WSC, water‐soluble carbohydrate.

C, control, no additive; Lp, Lactobacillus plantarum; Pp, Pediococcus pentosaceus; Ef, Enterococcus faecalis; LpPp, combination of Lactobacillus plantarum and Pediococcus pentosaceus; LpEf, combination of Lactobacillus plantarum and Enterococcus faecalis; LpPpEf, combination of Lactobacillus plantarum, Pediococcus pentosaceus, and Enterococcus faecalis.

^a‐e^Means within a column without a common superscript letter differ.

The LA/AA ratio is generally used as one of the indicators of silage fermentation (Trabi *et al*., 2017). In this study, the highest LA/AA ratio was observed in the Pp‐inoculated silage after one day of fermentation, as well as in the LpPp‐inoculated silage after 3–60 days of ensiling (Table 1). It might be because, as a coccus‐shaped LAB, Pp could initiate lactic acid fermentation to reduce pH at the initial stage of ensiling, which in turn stimulated the growth of *Lactobacillus* (McDonald *et al*., 1991). In this case, Lp grew more vigorously in the low pH environment to produce lactic acid in the LpPp‐inoculated silage. Accordingly, the lowest LA/AA ratio was observed in the Ef‐inoculated silage. However, Li *et al*. (2018) reported that a higher lactic acid concentration and LA/AA ratio were found in the silage inoculated with Ef compared with the control, and they speculated that Ef could metabolize more fermentable substrates via biodegradation of structural carbohydrates. Here, the fibre content in the Ef‐inoculated silage was also decreased compared with the control, but it did not accelerate the accumulation of lactic acid.

The DM content was greater in the Lp‐inoculated silage compared with other groups, and the lowest DM content was observed in the LpPpEf‐inoculated silage (Table 2). DM loss increased in the LpPpEf‐inoculated silage whereas it decreased in the Lp‐, Pp‐ and LpPp‐inoculated silages as compared with the control (Table 2). One of the most undesirable anaerobic fermentation processes is the conversion of lactic acid to butyric acid in silage, which causes a 51% loss of DM (Muck, 2010). However, there was no butyric acid detected in any groups in this study. In contrast, the metabolism of yeasts always leads to DM loss during ensiling, which utilizes soluble carbohydrates (Ávila *et al*., 2014). In the present study, the rapid decrease in pH value of the Lp‐, Pp‐ and LpPp‐inoculated silages as soon as fermentation was initiated probably inhibited the activity of yeasts, thus decreasing the DM loss in Lp‐, Pp‐ and LpPp‐inoculated silages. Ellis *et al*. (2016) reported that the rapid reduction of pH in the first 3 days of fermentation was important to inhibit undesirable microorganism growth and decrease nutrient loss in the silage. The NPN contents in the LAB‐inoculated silages decreased compared with the control, and the lowest NPN content was observed in the LpPp‐inoculated silage (Table 2). A silage with more true protein nitrogen rather than NPN can improve the efficiency of rumen microbial‐N synthesis (Pahlow *et al*., 2003). Therefore, the silages inoculated with Lp, Pp or LpPp were more favourable to forage N utilization by ruminants. The NH_3_‐N concentration is often used to indicate protein breakdown in silages (Pahlow *et al*., 2003). The synergistic effect of plant protease activity and microbial activity typically causes NH_3_‐N production in silages. Clostridium and plant proteolytic enzymes are active when the pH is between 5 and 6 (Wang *et al*., 2019b). Therefore, the lower pH inhibited the activity of plant proteases and the growth of *Enterobacteria* and *Clostridium* in silages inoculated with Lp, Pp and LpPp, thereby lowering the NH_3_‐N concentration in these silages. Although applications of Ef, LpEf or LpPpEf did not show benefits in nutrient preservation, they reduced the fibre content in ensiled alfalfa. Li *et al*. (2018) also reported that *E. faecalis* isolated from Tibetan yak rumen had the ability to decrease the aNDF and ADF contents of *Pennisetum sinese* silage. The strain *E. faecalis* AH38 was isolated from alfalfa silage after 7 days of ensiling based on its characteristics of better growth and good acid production ability in this study. However, whether the strain has cellulose‐degrading ability needs further confirmation.

### Bacterial communities and dynamics in alfalfa silage

**Fig. 1:**
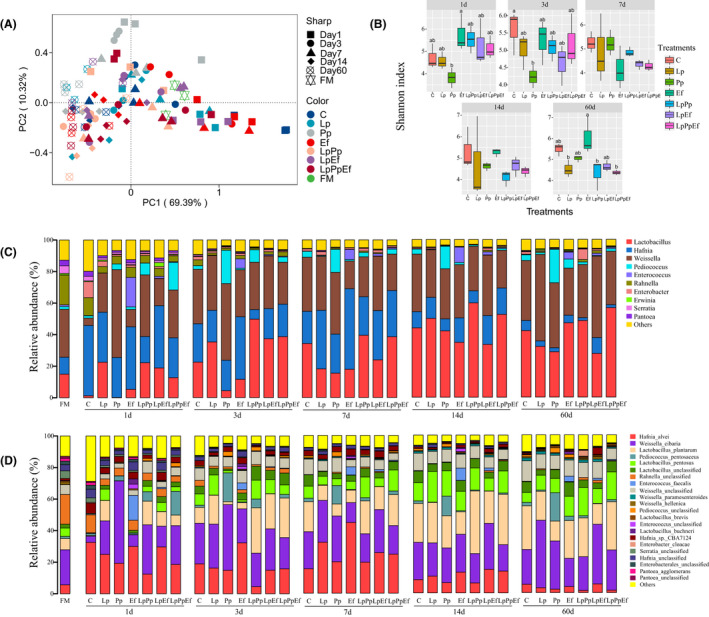
Microbial community dissimilarities and diversities in alfalfa silage during ensiling. C, Control (samples without inoculants); Lp, samples inoculated with *L. plantarum*; Pp, samples inoculated with *P. pentosaceus*; Ef, samples inoculated with *E. faecalis*; LpPp, samples inoculated with *L. plantarum* and *P. pentosaceus*; LpEf, samples inoculated with *L. plantarum* and *E. faecalis*; LpPpEf, samples inoculated with *L. plantarum*, *P. pentosaceus* and *E. faecalis*. Arabic number indicating days of ensiling. A. The community dissimilarities in different inoculant treatments and fermentation time, calculated via weighted UniFrac distances, with coordinates calculated using principal coordinates analysis (PCoA). B. The variations in community alpha‐diversities (Shannon index). C. Relative abundance of alfalfa silage bacterial genera across different inoculant treatments and fermentation time. D. Relative abundance of alfalfa silage bacterial species across different inoculant treatments and fermentation time.

Silage quality depends on the result of the competition between LAB and spoilage microorganisms, as well as the competition and collaboration between LAB (Ni *et al*., 2018). According to the PCoA analysis (Fig. 1A), obvious differences in the succession of bacterial communities in the different inoculants were observed during ensiling. In the early fermentation stage (1–3 days), the bacterial community in the Pp‐inoculated silage was clearly separated from the other groups. It might be because Pp rapidly initiated lactic acid fermentation and reduced the pH, then influenced the bacterial community succession. On the 60‐d fermentation, the bacterial communities in Lp‐, Pp‐ and LpEf‐inoculated silages were also separated from the other groups. The results of the dynamics of α diversity indicated that the Shannon index in the Pp‐inoculated silage was lower than the Ef‐inoculated silage fermented for 1 day and the control silage fermented for 3 day, respectively (Fig. 1B). This result was probably due to the lowest pH value obtained in the Pp‐inoculated silage at the fermentation times of 1 and 3 days, which rapidly created an acidic silage environment and consequently decreased bacterial diversity (Wang *et al*., 2020). After 60 days of ensiling, the Shannon indices were decreased in Lp‐, LpPp‐ and LpPpEf‐inoculated silages compared with the Ef‐inoculated silage. It might be because Lp grow vigorously in the late stage of ensiling due to its stronger acid resistance compared with cocci like Pp and Ef (Keshri *et al*., 2018). Wang *et al*. (2020) reported that *Enterococcus* could accelerate lactic acid fermentation during the early stage of fermentation; however, the Ef‐inoculated silage maintained a high pH value and bacterial diversity (Shannon index) during ensiling, which indicated that the Ef strain used in this study was less competitive than the epiphytic bacteria in alfalfa.

The dynamics of bacterial communities in alfalfa silage are shown in Fig. 1C (at the genus level) and Fig. 1D (at the species level). The main epiphytic species of fresh alfalfa were *Weissella cibaria* and *Rahnella‐unclassified*, followed by *Hafnia alvei*, *L. plantarum*, *L. pentosus* and unclassified *Weissella* (Fig. 1D). During the process of ensiling, *Lactobacillus*, *Hafnia* and *Weissella* were the most dominant genera in all groups before 7 days of ensiling; however, there was a sharp decrease in the relative abundance of *Hafnia* after 14 days of fermentation, therefore, *Lactobacillus* and *Weissella* became the predominant genera in all groups (Fig. 1C). It was reported that *Weissella* was considered as an early colonizer and then replaced by acid‐resistant *Lactobacilli* with a decrease in pH and the progress of fermentation of ryegrass silage (Graf *et al*., 2016). Interestingly, *Weissella* (*W. cibaria*) maintained a high relative abundance during the entire ensiling of alfalfa in the present study (Fig. 1C, 1D). That might be attributed to the stronger vitality and competitiveness of the epiphytic *Weissella* during the fermentation process in this study.

In the silages fermented for 1 day, the relatively high abundance of *Hafnia* was observed in the control, Ef‐ and LpEf‐inoculated silages, which might explain the higher pH value of those three groups after 1 day of ensiling. Interestingly, all the inoculants increased the relative abundance of *W. cibaria* relative to that in the control, and the highest abundance of *W. cibaria* was observed in the Pp‐inoculated silage (55.71%). This result suggested that Pp inoculation could accelerate the growth of epiphytic *W. cibaria* in the silage once fermentation was initiated. As expected, in silages fermented for 1 and 3 days, Pp‐ and Lp‐included inoculants increased the relative abundance of *P. pentosaceus* or *L. plantarum* in the treated silages compared with the control. However, the relative abundance of *E*. *faecalis* only increased in the silage inoculated with Ef alone. Therefore, it can be concluded that the strains Lp and Pp are more competitive than the strain Ef used in the present study, contrary to the report by Wang *et al*. (2020) that *Enterococcus* was often inoculated to enhance the fermentation quality of silage. In the 3‐d silages, all inoculants increased the relative abundance of *L. plantarum* except for the Pp‐ and Ef‐inoculated silages. In addition, the relative abundance of *P. pentosaceus* increased rapidly from 3.08% to 18.24% from 1 to 3 days of fermentation in the Pp‐inoculated silage, and then remained at a constant level until the end of fermentation. The relative abundance of *H. alvei* was still higher in all groups after 7 days of fermentation. *Hafnia*, a genus in *Enterobacteriaceae*, has proteolytic activities and can decarboxylate and deaminate some amino acids (Yuan *et al*., 2020). Therefore, *Hafnia* could contribute to the proteolysis in ensiled alfalfa at the early stage of fermentation, which may not be mainly caused by plant enzymes (Ding *et al*., 2013). The higher relative abundance of *Hafnia* in the Ef‐inoculated silage at the early phase of ensiling was consistent with the higher NH_3_‐N content in the Ef‐inoculated silage. After 14 days of fermentation, *L*. *plantarum* and *L pentosaceus* increased in all silages, and *L. plantarum* abundance was the highest in the LpPp‐inoculated silage fermented for 14 days, followed by the LpPpEf‐inoculated silage. This result indicates that *Pediococcus* could stimulate *Lactobacillus* growth (Yang *et al*., 2019).

**Fig. 2:**
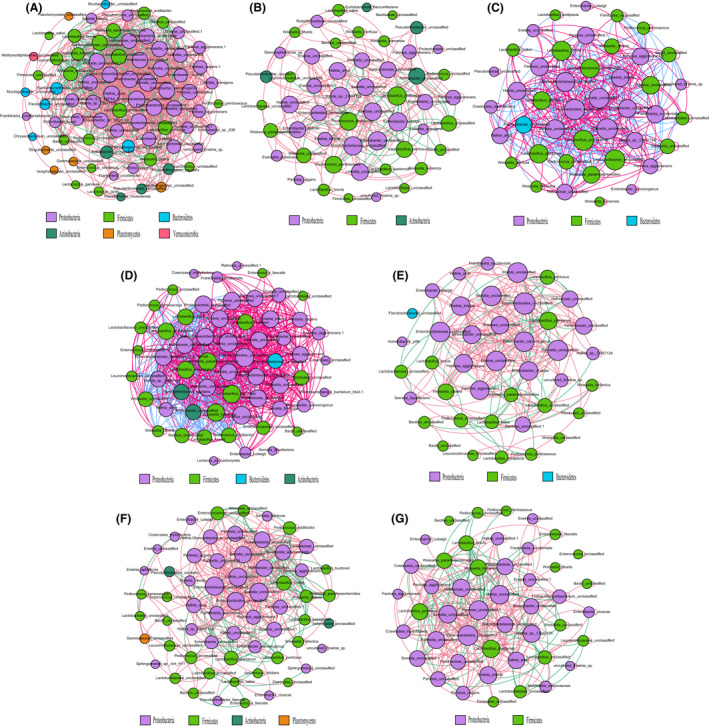
Interaction networks of the alfalfa silage microbiota. 16S rRNA gene‐based correlation network of the alfalfa silage microbiota is calculated from the bacteria with a relative abundance greater than 0.2%. Node size is scaled based on the overall abundance of each taxon in the microbiota. Edge width is proportional to the strength of association between each metabolite‐phylotype pair (as measured by the correlation), red edge indicates positive correlations and green edge indicates negative corrections. A. Control (samples without inoculants), (B) samples inoculated with *L. plantarum*, (C) samples inoculated with *P. pentosaceus*, (D) samples inoculated with *E. faecalis*, (E) samples inoculated with *L. plantarum* and *P. pentosaceus*, (F) samples inoculated with *L. plantarum* and *E. faecalis*, (G) samples inoculated with *L. plantarum*, *P. pentosaceus*, and *E. faecalis*.

The interactions between the microorganisms in the silage system are complex. In the current study, we used a microbial network to construct the correlations among species in the bacterial communities and to statistically identify the keystone taxa which had crucial effects on the microbial structure (Fig. 2). Our previous study showed that inoculation changed the correlation of bacterial communities, and the identified keystone taxa were completely different among the control, *L*. *plantarum*, and *L. buchneri‐*inoculated silages (Xu *et al*., 2020). Similarly, the present results also indicated that different inoculants and their combinations obviously altered the correlations within microbiota and the keystone species for modulating the fermentation process. It seems that more complicated correlations and interactions between bacterial species were observed in the control and Ef‐inoculated silages with relatively poor fermentation qualities. Contrarily, the network results showed that silages with a high fermentation quality had simplified bacterial correlation structures. Based on the network analysis, unclassified *Enterobacterales*, and *Klebsiella* in the control silage, *E. cloacae* and *L. plantarum* in the Lp‐inoculated silage, *L. pentosus* and unclassified *Proteobacteria* in the Pp‐inoculated silage, unclassified *Yersiniaceae* and *Proteobacteria* in the Ef‐inoculated silage, *L. plantarum* and *Pantoea agglomerans* in the LpPp‐inoculated silage, unclassified *Proteobacteria* and *Pantoea* in the LpEf‐inoculated silage, and *L. pentosus* and unclassified *Proteobacteria* in the LpPpEf‐inoculated silage were identified as the keystone taxa. The keystone taxa have considerable influence on the microbial community and function, but their abundances were not directly proportional to their effects (Banerjee *et al*., 2018; Xu *et al*., 2020). Confusingly, undesirable bacteria were identified as the keystone taxa in the control, Ef‐ and LpEf‐inoculated silages. Even so, poor fermentation quality was observed in these inoculated silages.

### Correlation analysis of the bacterial community and fermentation characteristics

**Fig. 3:**
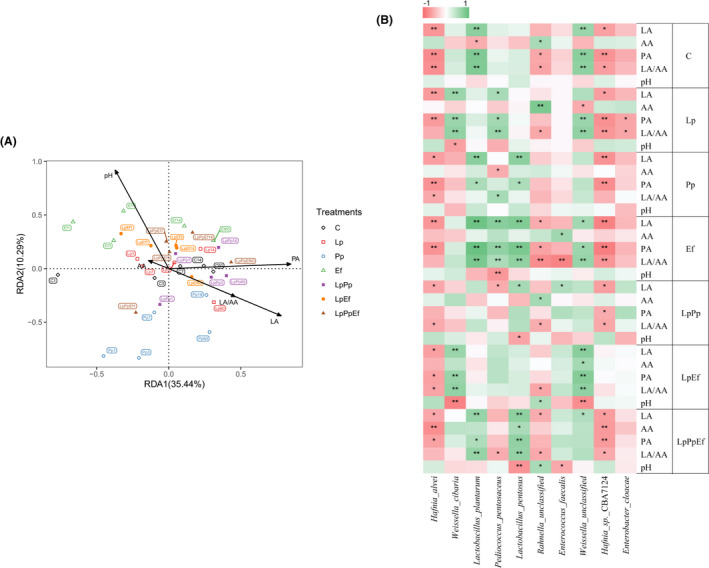
Correlation analysis of the bacterial communities with fermentation characteristics. C, Control (samples without inoculants); Lp, samples inoculated with *L. plantarum*; Pp, samples inoculated with *P. pentosaceus*; Ef, Samples inoculated with *E. faecalis*; LpPp, samples inoculated with *L. plantarum* and *P. pentosaceus*; LpEf, samples inoculated with *L. plantarum* and *E. faecalis*; LpPpEf, samples inoculated with *L. plantarum*, *P. pentosaceus* and *E. faecalis*. A. Redundancy analysis (RDA) plot showing the correlations between fermentation characteristics and bacterial community composition. The canonical axes are labelled with the percentage of total variance explained (%). Arrow lengths indicate the variance explained by fermentation characteristics. Different inoculant treatments at different fermentation times are presented as individual data points. Arabic numbers indicate days of ensiling. B. Association analysis between bacterial species and fermentation characteristics. Fermentation characteristics are displayed horizontally and the bacterial community information is displayed vertically. The corresponding value of the middle heat map is the Spearman correlation coefficient r, which ranges between − 1 and 1; *r* < 0 indicates a negative correlation (red), *r* > 0 indicates a positive correction (blue), and ‘*’ and ‘**’ represent *P* < 0.05 and *P* < 0.01, respectively.

In the current study, RDA analysis was used to assess the relationships between the fermentation characteristics and bacterial community composition (Fig. 3A), and the heatmap was used to assess the correlations between the bacterial species and fermentation characteristics (Fig. 3B). According to results of the RDA analysis, the combination of variables explained 49.87% of the bacterial community structure variances. The arrow representing acetic acid was shorter, indicating that acetic acid had less influence on the bacterial communities in all groups in this study. The lactic acid concentration and LA/AA ratio were found to be the determining factors in shaping the bacterial community of Pp‐, and LpPp‐inoculated silages during the entire ensiling process, whereas the bacterial community of the Ef‐inoculated silage during ensiling was substantially influenced by the pH value. The results were consistent with the fermentation characteristics in this study, as a higher concentration of lactic acid was seen in the Pp‐ and LpPp‐inoculated silages, and a higher pH value was observed in the Ef‐inoculated silage during ensiling. In addition, bacterial communities in the Lp‐, LpEf‐ and LpPpEf‐inoculated silages after 60 days of ensiling were influenced by lactic acid concentration and LA/AA.

We also performed a correlation analysis between the relative abundances of the top 10 species and the fermentation characteristics of the silages (Fig. 3B). As expected, *H. alvei* and *H. sp*. CBA7124 were negatively correlated with the concentrations of lactic acid and propionic acid, and LA/AA ratio in all groups, whereas *Lactobacillus*, *Weissella* and *Pediococcus* were positively correlated with the concentrations of lactic acid and propionic acid, and LA/AA. However, the species of microorganisms that were positively related to fermentation characteristics were different (Fig. 3B). The present results showed that different silage inoculants and their combinations had different effects on fermentation characteristics, and the inoculants might not directly affect fermentation characteristics. Guo *et al*. (2018) found that alfalfa silage inoculated with *L. buchneri* could accelerate the growth of *L. plantarum* to some extent, resulting in a rapid decrease of the silage pH. It is interesting that *E. faecalis* was positively correlated with the acetic acid concentration and negatively correlated with LA/AA in the Ef‐inoculated silage. This might be due to the higher pH in the Ef‐inoculated silage. However, it is difficult to explain that lactic acid concentration was positively correlated with *E. faecalis* in the LpPp‐inoculated silage because a rather low relative abundance of *E. faecalis* was detected in this group.

### Functional shifts of bacterial communities in alfalfa silage

**Fig. 4:**
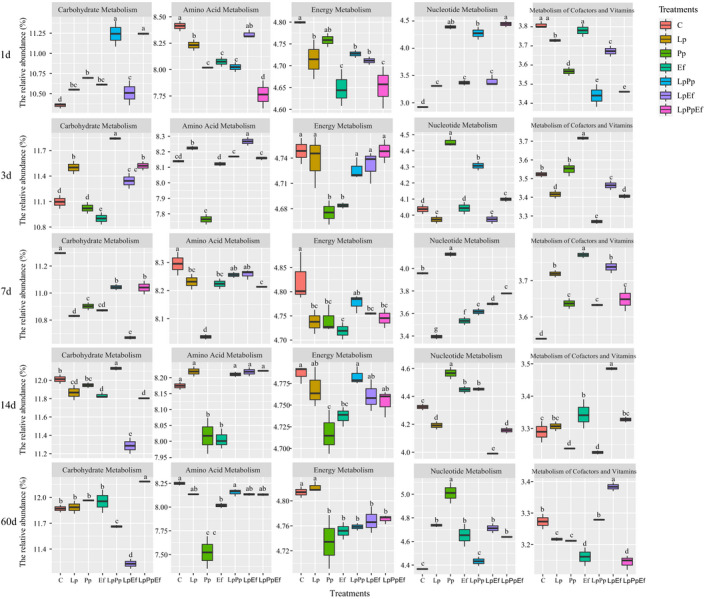
Microbial alterations contribute to functional shifts after fermentation with different inoculants and their combinations. C, Control (samples without inoculants); Lp, samples inoculated with *L. plantarum*; Pp, samples inoculated with *P. pentosaceus*; Ef, samples inoculated with *E. faecalis*; LpPp, samples inoculated with *L. plantarum* and *P. pentosaceus*; LpEf, samples inoculated with *L. plantarum* and *E. faecalis*; LpPpEf, samples inoculated with *L. plantarum*, *P. pentosaceus*, and *E. faecalis*. Summary of significant functional shifts predicted using Phylogenetic Investigation of Communities by Reconstruction of Unobserved States (PICRUSt). For each KEGG pathway, the second level of the predicted functional shift is shown with respect to the fermentation processes and inoculant treatments. ^a‐e^Indicates significant differences between inoculant treatments with the same silage period at *P* < 0.05.

The fermentation process in silage is mediated by microbial activities through complicated metabolic pathways to degrade substrates or transform metabolites. Functional prediction of bacterial communities allows us to assess the effect of bacterial communities on the changes in the metabolic pathways underlying silage formation. Therefore, we used the KEGG pathway database with PICRUSt to predict the metabolic pathways of silages treated with different bacterial inoculations and their combinations (Fig. 4). Xu *et al*. (2020) reported that the metabolic pathways related to silage fermentation were metabolism of carbohydrates, amino acid, energy and cofactors, and vitamins. Therefore, we chose these metabolic pathways, including nucleotide metabolism, for statistical analysis. In the present study, amino acid metabolism was lowest in the Pp‐inoculated silage from 3 days until the end of ensiling, which was consistent with the lowest pH and the reduction of NH_3_‐N in the Pp‐inoculated silage. Therefore, the lowest pH in the Pp‐inoculated silage inhibited the amino acid metabolism caused by undesirable microbes such as *Hafnia*, *Clostridium* and *Enterobacter* in the silage (Flythe and Russell, 2004; Yuan *et al*., 2020). Interestingly, the metabolism of cofactors and vitamins increased in the Ef‐inoculated silages fermented for 1, 3 and 7 days, and in the LpEf‐inoculated silages ensiled for 14 and 60 days, respectively. It could be deduced that *E. faecalis* might directly produce vitamins or accelerate vitamin production during ensiling. Our previous study found that higher concentrations of α‐tocopherol and β‐carotene were observed in silage inoculated with *P. acidilactici* J17 as compared with the control (Zhang *et al*., 2020). The expression of carbohydrate metabolism pathway was related to the relative abundance of total LAB in the bacterial community. Higher relative abundances of carbohydrate metabolism were observed in the LpPp‐inoculated silage during the early phase of ensiling (1 and 3 days), in control silage fermented for 7 days, in the LpPp‐inoculated silage ensiled for 14 days and in the LpPpEf‐inoculated silage ensiled for 60 days. Correspondingly, the higher relative abundances of total LAB in the bacterial communities were also observed in the above treatments compared with the other silages (Fig. 1D). Conversely, low relative abundances of carbohydrate metabolism were observed in silages with a lower relative abundance of total LAB in their bacterial communities. According to Pessione *et al*. (2010), amino acid decarboxylation, malate decarboxylation and arginine deimination are the three main energy metabolism routes in LAB. These routes lead to lactic acid accumulation during fermentation. Here, the relative abundance of energy metabolism was higher in the control and lower in Pp‐ and Ef‐inoculated silages during the entire ensiling period, which was inconsistent with the report of Xu *et al*. (2020) which energy metabolism was predicted to be upregulated in the inoculated silages in the middle stage of fermentation. The abundance of nucleotide metabolism was higher in the Pp‐inoculated silages compared with other groups after 3 days of ensiling, which were opposite of the results obtained in the abundance of energy metabolism. According to Kilstrup *et al*. (2005), nucleotides are substrates for DNA synthesis and serve as a major energy donor for cellular processes. It is therefore necessary to use other omics approaches like proteomics and metabolomics to further study the functions of the bacterial community during the ensiling of forages.

In summary, by using single‐molecule real‐time (SMRT) sequencing technology, we figured out the effects of different inoculants on the complicated biological process of the alfalfa ensiling ecosystem in terms of the succession of bacterial community, their interactions and metabolic pathways. The inoculants used in the present study altered the dynamics of bacterial community and the keystone taxa in different manners, and also dramatically changed the carbohydrate, amino acid, energy, nucleotide and vitamin metabolism of bacterial communities during ensiling. Silages with a high fermented quality (Lp‐, Pp‐ and LpPp‐inoculated silages) had simplified bacterial correlation structures, and the desirable bacterium *L. plantarum* was identified as the keystone taxa in Lp‐ and LpPp‐inoculated silages, and *L. pentosus* was the keystone taxa in Pp‐inoculated silage. However, the strain of Ef used in this study was not recommended as silage starter cultures indicated by higher NH_3_‐N concentration, poor fermentation quality and upregulated amino acid metabolism in inoculated silages. Therefore, the present results not only revealed the biological process underlying silage fermentation, but also may provide important information for developing target‐based regulation inoculants to produce high‐quality silage for animal production.

## Experimental procedures

### Lactic acid bacteria strains

In this study, the LAB strains used as silage inoculants were *L. plantarum* MTD‐1 (Lp; Vita Plus, Madison, MI, USA), *P. pentosaceus* (Pp, Vita Plus) and *E. faecalis* AH38 (Ef). The strain *E. faecalis* AH38 was isolated and screened from alfalfa silage after 7 days of ensiling in our laboratory due to its better growth performance and remarkable acid production ability.

### Alfalfa silage preparation

Alfalfa was grown for two years in an experiment plot located in Bengbu City, Anhui province, China (32°57′N, 117°12′E) and harvested in the late bud to early bloom stage in May 2019. The average soil OM in the cropping field was 11.1 g kg^‐1^; effective N, P and K in the soil were 82.9, 19.0 and 115.0 mg kg^‐1^, respectively. Fresh alfalfa was harvested by hand from four randomly selected sites (used as replication for each treatment) within a field of about 0.1 hectare (an experimental plot of alfalfa variety), taken into the laboratory immediately, and wilted to a dry matter (DM) content of 311.9 g kg^‐1^ of fresh weight (FW). The content of crude protein (CP) in alfalfa before ensiling was 218.7 g kg^‐1^ DM, neutral detergent fibre (αNDF) was 315.9 g kg^‐1^ DM, acid detergent fibre (ADF) was 223.4 g kg^‐1^ DM, water‐soluble carbohydrate (WSC) was 49.9 g kg^‐1^ DM, and the pH was 6.12. Before ensiling, the wilted forage was manually chopped into lengths of 1–2 cm by paper cutters. For each of the 4 locations, there were 35 piles of forage (7 inoculants treated‐plies for each fermentation time of 1, 3, 7, 14 and 60 days). The forage piles from each location were then treated separately with distilled water (control, C), *L. plantarum* (1 × 10^5^ CFU g FW^‐1^, Lp), *P. pentosaceus* (1 × 10^5^ CFU g FW^‐1^, Pp), *E. faecalis* (1 × 10^5^ CFU g FW^‐1^, Ef), a combination of *L. plantarum* (5 × 10^4^ CFU g FW^‐1^) and *P. pentosaceus* (5 × 10^4^ CFU g FW^‐1^) (LpPp), a combination of *L. plantarum* (5 × 10^4^ CFU g FW^‐1^) and *E. faecalis* (5 × 10^4^ CFU g FW^‐1^) (LpEf), and a combination of *L. plantarum* (3.3 × 10^4^ CFU g FW^‐1^), *P. pentosaceus* (3.3 × 10^4^ CFU g FW^‐1^) and *E. faecalis* (3.3 × 10^4^ CFU g FW^‐1^) (LpPpEf). The application rate (1 × 10^5^ CFU g FW^‐1^) of the inoculants used in each treatment was the least effective dosage for silage inoculation based on the previous study (Huisden *et al*., 2009). The detailed procedures of adding LAB inoculants and constructing laboratory‐scaled silos were described in our previous report (Zhang *et al*., 2020). The silage bags were stored at an ambient temperature (~ 25°C) for 60 days.

### Chemical and fermentation profile analyses

The silage bags were opened after 1, 3, 7, 14 and 60 days of ensiling. Fresh and silage samples (20 g) were blended with distilled water (180 ml) at a high speed for 30 s, followed by filtration via four layers of cheesecloth. The pH of the supernatant was measured using a glass electrode pH metre (PHSJ‐3F, CANY, Shanghai, China). Concentrations of lactic, acetic and propionic acids were determined according to the methods described by Zhang *et al*. (2020). The contents of non‐protein nitrogen (NPN), ammonia nitrogen (NH_3_‐N) and water‐soluble carbohydrates (WSC) were tested according to the method described by Licitra *et al*. (1996) and Ke *et al*. (2017). The chemical compositions of the silage were presented based on their DM base. Fresh and ensiled forage were dried at 55°C for 72 h for DM content measurement, and then ground with a mill for nutrient analyses (Ke *et al*., 2017). DM loss was calculated as the DM difference of the silages before ensiling and after 60 days of ensiling. The contents of CP, aNDF and ADF were determined according to Ke *et al*. (2017).

### Bacterial composition SMRT analyses

The bacterial total DNA extraction of fresh alfalfa and silages fermented for 1, 3, 7, 14 and 60 days were performed according to the description of Guo *et al*. (2018). Among the four replicates of each treatment, three replicated samples with the most similar pH values were chosen for DNA extraction. Construction of a 16S rRNA library, detection of DNA concentration and quality, and DNA purification for PCR amplification were conducted according to the methods of Guo *et al*. (2018). PCR amplification for SMRT sequencing was conducted according to the methods of Xu *et al*. (2019).

Analysis of the sequencing data, including sequence extraction and filtering, sequence optimization, taxa notes and alpha diversity calculation, was performed according to the report of Xu *et al*. (2019). Sample ordination based on the beta diversity was examined using the principal coordinate’s analysis (PCoA). Microbial networks were used to statistically calculate the correlations between predominant taxa; meanwhile, identification of keystone taxa in the bacterial communities was performed by the combined score of low betweenness centrality, high closeness centrality and high mean degree (Berry and Widder, 2014). Redundancy analysis (RDA) was performed to assess the relationship between the fermentation characteristics and bacterial communities (Dong *et al*., 2017). Heatmap analysis was performed to identify the correlation between the relative abundances of the silage bacteria species and the fermentation characteristics. Microbial functions were proof checked from the Kyoto Encyclopedia of Genes and Genomes (KEGG) database using Phylogenetic Investigation of Communities by Reconstruction of Unobserved States (PICRUSt).

### Statistical analyses

The experimental protocol had a 7 × 5 factorial design with seven inoculants and five ensiling times. The data for pH and organic acids were analysed using the general linear model procedure of the Statistical Package for Social Science (SPSS 21.0; SPSS, Inc., Chicago, IL, USA) according to the model:

$$Y_{\mathrm{ijk}}=\mu +T_{i}D_{j}+\left(T\times D\right)ij+\varepsilon_{\mathrm{ijk}},$$

where *Y*_ijk_ represents the response variable, μ is the overall mean, *T_i_* is the effect of inoculants, *D_j_* is the effect of ensiling time, (*T* × *D*)*ij* is the effect of the interaction between the inoculants and ensiling time, and *ε_ijk_* is the residual error. Means were separated by Tukey’s test. Chemical compositions of 60‐days silage samples were analysed using one‐way ANOVA. Tukey’s test was also used for pair‐wise mean comparisons. Significance was considered at *P* < 0.05, and the tendency was declared at *P* < 0.1.

## Funding Information

This work was funded by the National Key R&D Program of China (project no. 2017YFE0104300) and the National Natural Science Foundation of China (project no. 31672487).

## Conflict of interest

None declared.

## Data Availability

Raw sequencing files and associated metadata have been deposited in NCBI’s Sequence Read Archive (accession PRJNA664087), https://www.ncbi.nlm.nih.gov/sra.
